# Editorial: Women in genitourinary oncology: 2021

**DOI:** 10.3389/fonc.2022.1027852

**Published:** 2022-09-28

**Authors:** Sanja Štifter

**Affiliations:** Department of Pathology, Aarhus University Hospital, Aarhus, Denmark

**Keywords:** women, genitourinary oncology, prostate carcinoma, renal cell carcinoma, penis carcinoma

Recently I had the privilege to edit a Frontiers Topic dedicated to women in genitourinary oncology research and practice. Exquisite women researchers helped us promote topics from genitourinary oncology together with their peers. The aim was to gather a collection of state-of-the-art research articles showing that women in research have a valuable position not only as a part of a team but also as leading researchers or group leaders. I can confidently say that this particular topic attracted a very interesting group of articles covering a broad spectrum of the genitourinary oncology field (**Figure 1**).

**Figure 1:**
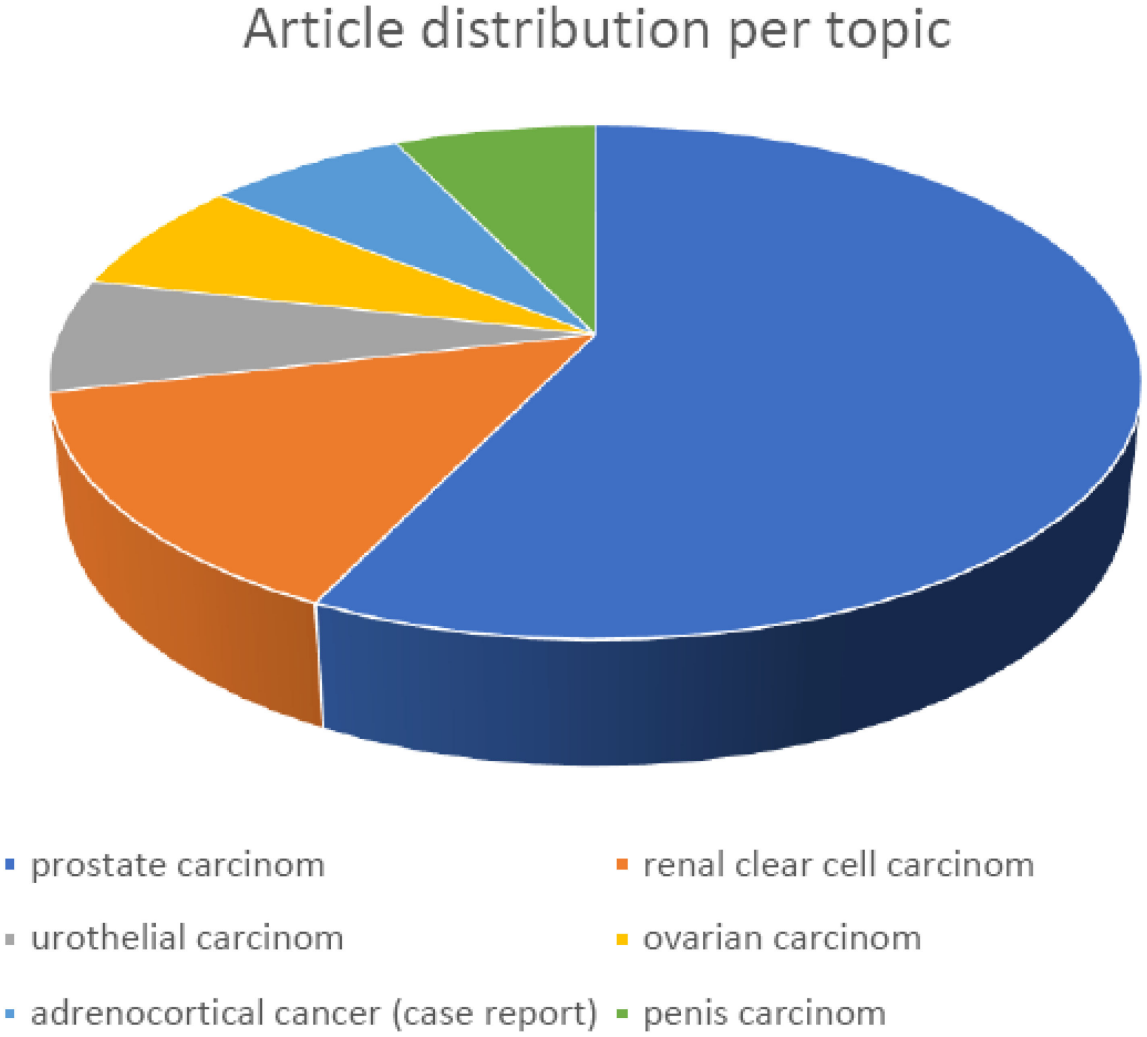
Diagram represents article distribution per topic showing the most frequent articles discussing prostate and renal cell clear cell carcinoma.

As it was stated in the topic introduction, at present, fewer than 30% of researchers worldwide are women. Therefore, this topic is even more relevant in strengthening efforts to overcome gender stereotypes discouraging girls and women away from science-related fields, and STEM research in particular.

Editing this research Topic was for me a very positive experience concluded with a collection of articles mostly covering prostate cancer oncology research and clinical practice. Different prostate cancer therapeutic strategies were well presented in several articles by groups of authors including Raju et al. and Maggi et al. Raju et al concluded by presenting a real-world data regional study that showed both abiraterone and enzalutamide will remain standard-of-care treatments in Australian men with metastatic castration-resistant prostate cancer (mCRPC), as the survival and disease control benefits of these agents have continued to be seen in numerous real world studies, consistent with the phase III clinical trials.

New emerging therapeutic targets with preliminary results were presented in articles by Marvaso et al. and Masson et al. It was very interesting to observe a substantially high interest in the correlation between psychosocial stress and age-influenced depression and anxiety-related behavior driving tumor inflammatory cytokines and accelerating prostate cancer growth in mice by Bellinger et al. A clinical prospective study presented by Logozzi et al., which showed that plasmatic exosome number and size distinguish prostate cancer patients from healthy individuals, gained great attention from audiences.

A systematic review and meta-analysis of combined therapy for renal cell carcinoma, presented by a group of authors led by Tao et al. gave an interesting perspective on balancing the risk-benefit ratio. In volume two, a mini-review on targeting strategies in the treatment of fumarate hydratase deficient renal cell carcinoma provides an authentic review of therapeutic approaches presented by a group of researchers led by Renate Pichler.

Results of a multicenter prospective study presented quality-of-life outcomes in female patients with ileal conduit or orthotopic neobladder urinary diversion by Siracusano et al.

Another interesting piece of clinical research was presented by Xiong et al., where a population-based study analyzed the prevalence and outcomes of unilateral versus bilateral oophorectomy in women with ovarian cancer.

It is not often that a Research Topic accepts a case report, but in this collection, we found one case report of interest by Tőke et al. describing complete remission of advanced adrenocortical cancer following mitotane monotherapy which, besides presenting a case report, gives a thorough literature review of predictive markers.

Finally, a very interesting original research article elucidating the patterns of treatment and outcomes in older men with penile cancer, A SEER Dataset Analysis, is part of volume two presented by Maria T. Bourlon et al.

Instead of a conclusion, I would like to point out that such platforms promoting the work of women scientists, across all fields of Oncology, are beneficial in giving more visibility to women researchers. This can be inspiring for young girls and women and, at the same time, ensures sustainable development of science too. Gender equality must be promoted as well as gender stereotypes defeated, and girls and women should be encouraged to pursue STEM careers.

I would like to dedicate this editorial to my recently departed colleague, Professor Ondrej Hess, who was an outstanding researcher, teacher, pathology expert, and nature supporter and also a strong advocate of gender equality in science and research.

## Author contributions

The author confirms being the sole contributor of this work and has approved it for publication.

## Conflict of interest

The author declares that the research was conducted in the absence of any commercial or financial relationships that could be construed as a potential conflict of interest.

## Publisher’s note

All claims expressed in this article are solely those of the authors and do not necessarily represent those of their affiliated organizations, or those of the publisher, the editors and the reviewers. Any product that may be evaluated in this article, or claim that may be made by its manufacturer, is not guaranteed or endorsed by the publisher.

